# Modeling Mechanochemical Depolymerization of PET in Ball-Mill Reactors Using DEM Simulations

**DOI:** 10.1021/acssuschemeng.3c06081

**Published:** 2024-06-04

**Authors:** Elisavet Anglou, Yuchen Chang, William Bradley, Carsten Sievers, Fani Boukouvala

**Affiliations:** † School of Chemical & Biomolecular Engineering, Georgia Institute of Technology, Atlanta , Georgia 30332, United States; ‡ Renewable Bioproducts Institute, Georgia Institute of Technology, Atlanta, Georgia 30332, United States

**Keywords:** ball milling, DEM, computer vision, plastics recycling, mechanochemistry

## Abstract

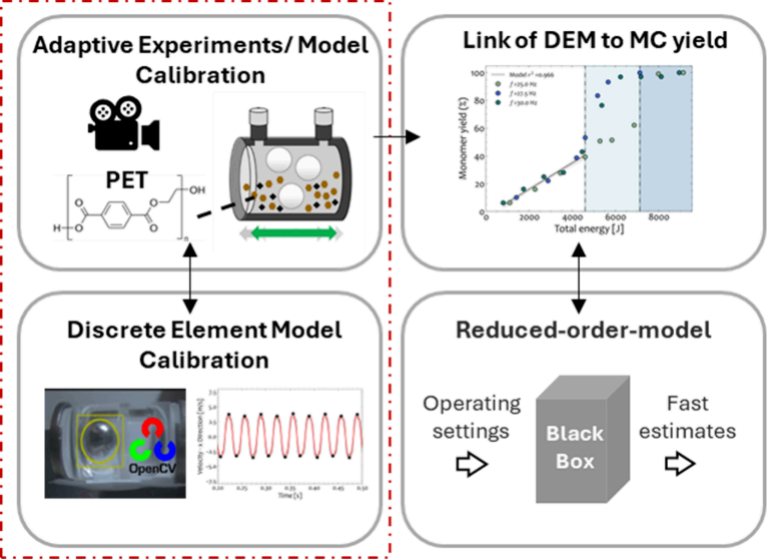

Developing efficient and sustainable chemical recycling pathways for consumer plastics is critical for mitigating the negative environmental implications associated with their end-of-life management. Mechanochemical depolymerization reactions have recently garnered great attention, as they are recognized as a promising solution for solvent-free transformation of polymers to monomers in the solid state. To this end, physics-based models that accurately describe the phenomena within ball mills are necessary to facilitate the exploration of operating conditions that would lead to optimal performance. Motivated by this, in this paper we develop a mathematical model that couples results from discrete element method (DEM) simulations and experiments to study mechanically-induced depolymerization. The DEM model was calibrated and validated via video experimental data and computer vision algorithms. A systematic study on the influence of the ball-mill operating parameters revealed a direct relationship between the operating conditions of the vibrating milling vessel and the total energy supplied to the system. Moreover, we propose a linear correlation between the high-fidelity DEM simulation results and experimental monomer yield data for poly(ethylene terephthalate) depolymerization, linking mechanical and energetic variables. Finally, we train a reduced-order model to address the high computational cost associated with DEM simulations. The predicted working variables are used as inputs to the proposed mathematical expression which allows for the fast estimation of monomer yields.

## Abstract 2

We develop and experimentally validate a modeling framework for the mechanochemical depolymerization of poly(ethylene terephthalate) in a ball mill, linking operating conditions to reaction yield.

## 1 Introduction

Industrialized economies have traditionally relied on linear manufacturing processes in which raw materials are transformed into useful products and later discarded as waste.^1−3^ Yet, this economic structure has a substantial impact on natural resource depletion and the environment, particularly in the case of plastics. Three hundred sixty million tons of plastics were manufactured worldwide in 2018, but only 10% were recycled, while the majority (80%) ended up in landfills or the oceans.^3,4^ Furthermore, the degradation of plastic waste into microplastics and hazardous water-soluble compounds endangers human and animal health.^2,5^ Therefore, addressing the negative environmental consequences of plastic waste management has stimulated great interest towards a circular economic model in which waste materials, such as plastics, will be recycled back into the economy and will be remanufactured into useful products.

Plastic waste recycling methods can be categorized into preconsumer, mechanical recycling, chemical recycling, and energy recovery pathways.^6−8^ Currently, the majority of recycling infrastructures rely on mechanical recycling and waste-to-energy processes. The distinct difference between those two methods is the final product: recycled plastic versus energy. Energy recovery methods refer to burning down waste to produce energy. This can be a sustainable solution, especially for mixed plastics that are hard to separate and/or recycle. However, in most cases, waste-to-energy routes hinder circularity and reusability of plastics. On the contrary, in mechanical recycling, the plastic waste is physically molded into new plastic products,^9^ allowing for multiple uses of the same material into the production chain. However, the mechanical and thermal degradation of the polymer during processing compromise the integrity and quality of recycled plastic products.^10^ As a result, each plastic product can be recycled a limited number of times and only if mixed with large quantities of virgin polymers.^3,11^ Chemical recycling routes have recently emerged as promising methods to directly depolymerize polymers into their monomeric molecules, bypassing material degradation issues of mechanical recycling.^12^ Particularly for the case of PET waste, chemical recycling depolymerization routes focus on hydrolysis, glycolysis, alcoholysis, and ammonolysis reactions.^13^ Typically, such processes operate at extreme conditions (e.g., high temperatures and/or pressures) and use large amounts of solvents, thereby hindering their economic viability. Thus, the development of alternative processes for plastic waste is crucial for implementing sustainable practices and reducing the negative environmental impacts.

One promising alternative is the depolymerization of polymers in the solid state via mechanically-induced reactions.^14−16^ Mechanochemical reactions are typically performed in ball mills, in which contacts and collisions between grinding surfaces (balls and reactor wall) supply the energy required to chemically transform the (usually particulate) solid reactants caught between these surfaces.^15,17−20^ Mechanochemistry has been successfully demonstrated on a laboratory scale for the production of lignocellulosic biomass,^21,22^ cellulose,^23−25^ ammonia^26−29^ and lignin.^30−33^ Particularly for poly(ethylene terephthalate) (PET), Štrukil^34^ and Tricker et al.^3^ recently demonstrated its complete depolymerization to monomers inside ball mills. Moreover, mechanochemical routes have recently been explored for the depolymerization of various polymers such as polystyrene (PS),^35,36^ polyethylene (PE),^37^ and poly(α-methylstyrene) (PMS)^27^ and in the dechlorination of polyvinyl chloride (PVC).^38,39^ In addition to the ability to efficiently process solid reactants, ball milling is a highly scalable industrial process being utilized in a wide variety of grinding applications, from minerals and cement, to chemicals and pharmaceuticals.^40−44^ Despite these advantages, mechanochemical reactions are often seen and modeled as “black-boxes”, which hinders the fundamental understanding of mechanically induced reactions.^45^ In attempts to model mechanochemical reactions, semiempirical models have been proposed across various branches of mechanochemistry.^17,18,28,46,47^ However, these models are often limited by extrapolation issues, which restrict their utility in exploring conditions such as reactor geometry or grinding media material that would lead to optimal performance. Therefore, computational frameworks that would enable accurate predictions under other conditions are necessary to realize the use of mechanochemistry for waste processing.

Mathematical modeling of ball mills has been extensively explored over the last 70 years, starting from empirical correlations^48^ and semiempirical population balance models,^40,43^ to high-fidelity discrete element method (DEM) models.^40^ First proposed by Cundall and Strack,^49^ DEM models have received considerable attention due their ability to describe the complex kinematics of moving entities and thus have been successfully used across various applications including the development of kinetic models for mechanochemical reactions.^39,50^ In DEM, the position and energetics of each discrete entity are evaluated over short time scales considering the effect of the surrounding population and geometry to the forces acting on each entity.^40,51^ Parameters such as the geometry, material properties, and processing conditions are necessary to develop accurate digital-twin models.^52^ Thus, DEM simulations provide a means to obtain a first-principles understanding of the ball milling grinding efficiency and the influence of mechanical factors on the performance of mechanochemical reactions. Although DEM models are very powerful and can replicate the dynamic behavior of a system, several limitations exist regarding (a) the material parameter calibration that may require expensive experimental setups; (b) the high computational cost associated with the numerical techniques needed to simulate movement of discrete entities with small timesteps; and (c) the particle shapes that are often approximated as spheres, a choice that is not always accurate.^53,54^

The calibration of the DEM material parameters significantly influences the prediction of the model. Material properties, such as the Young’s modulus, material densities, and other mechanical properties, are measured experimentally and are used as the inputs to the DEM simulation.^52^ Two main approaches have been proposed to calibrate the DEM material parameters, namely, the direct and bulk measurement methods.^52,55,56^ In the direct measurement approach, specialized experimental setups are used to obtain the values of the material properties required as inputs to the DEM simulation. Although these measurements are accurately obtained, the required experimental methods, such as the direct shear or particle impact experiments, can be very expensive.^40,52^ In contrast, in the bulk measurement approach, the values of the material parameters are adjusted to match the DEM simulation with experimentally observed features.^55,56^ Multiple authors have used different methodologies to calibrate their DEM material parameters via the bulk measurement approach. Most commonly employed experiments are the drop test,^57^ the angle-of-repose test,^52,53^ and the ring shear test,^58^ which provide data that can be used to adjust the material parameters of the simulation to mimic the observed system. More advanced examples include high-speed filming of the dynamic operation,^59,60^ which lead to collection of large, dynamic data sets.

Another challenge associated with DEM models is the high computational cost required to run a simulation where hours, days, or months may be required even when supercomputers are available. To address this, surrogate models have been successfully employed to translate DEM process inputs to outputs.^61−63^ Rogers and Ierapetritou^64^ used a Kriging surrogate model to represent velocity profiles from DEM simulation in blending application. In Metta et al.,^51^ mechanistic data obtained from DEM simulations were mapped using Kriging and artificial neural network (ANN) surrogate models for a milling process, while in Barrasso et al.,^65^ collision frequency from the DEM simulations are used as inputs into a population balance model using an ANN surrogate framework. The main benefit of developing surrogates is that once trained, they can be used to interpolate simulation results for various operating conditions for which the expensive simulations were not run (in this case DEM). Thus, they have played a significant role in connecting computationally expensive models with optimization algorithms as they provide a means to accurately represent simulation outputs in a fast manner.^66−68^

**Figure 1:**
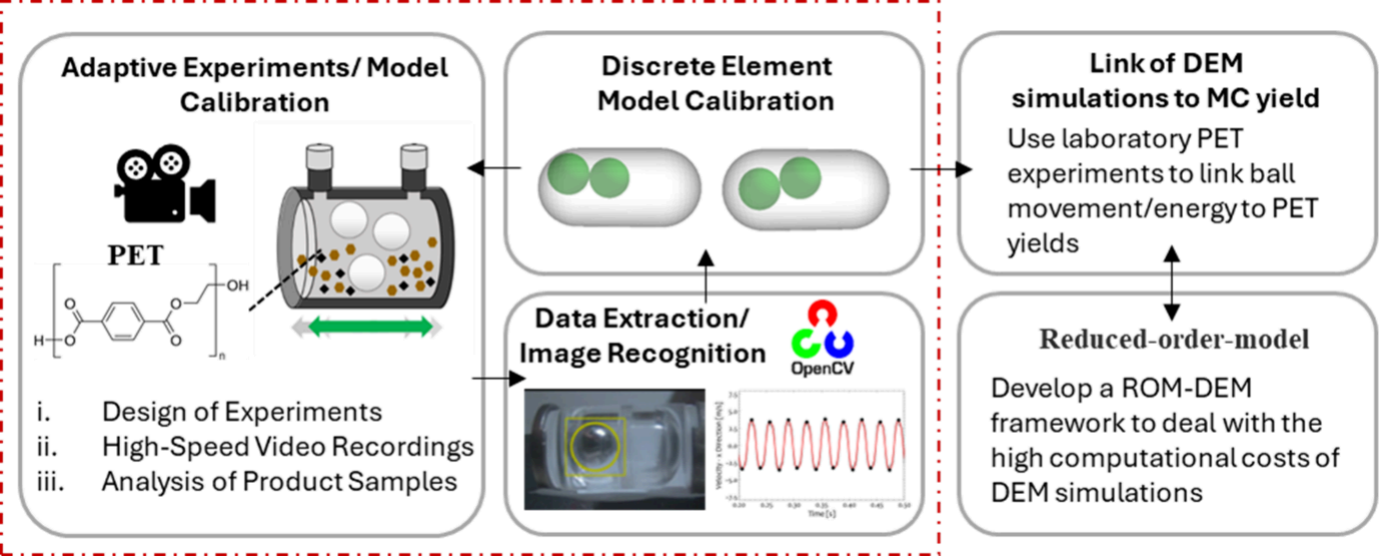
Main components of our study include experimental design, machine learning computer vision tools, and development of a discrete element method model for the lab-scale reactor. When coupled with parameter estimation, we use all components to develop correlations for a mechanochemical ball-mill reactor. A reduced-order-model (ROM) is employed to translate DEM inputs to outputs with reduced computational cost.

The present work is motivated by the need for an accurate and efficient mathematical representation of reactive ball milling to aid the process design and optimization for mechanochemical recycling of plastic waste. The use of high-fidelity DEM models is proposed as a means to explore the efficiency of the mechanochemical processing of PET waste via first principles. Following this idea, a multiscale modeling framework that includes DEM models and computer vision (CV) object detection algorithms for model validation, is utilized as a tool to describe the motion of the grinding bodies throughout the milling operation. Subsequently, the DEM simulation is exploited and linked with experimental results to construct efficient correlations that predict the mechanochemical depolymerization of PET. Finally, a surrogate model is trained to translate the mechanistic DEM inputs to outputs in an efficient manner to provide fast estimates of yields. An illustration of the steps and computational tools utilized to model the mechanochemical PET depolymerization is depicted in Figure 1.

The remainder of this paper is structured as follows. In Section 2, the DEM model is formulated and a general outline of the calibration and validation methodology is provided. The performance of the DEM model is explored in the Results section (Section 3). Section 4 highlights the mathematical link between the DEM simulations and the mechanochemical depolymerization experiments, while Section 5 discusses the development of a DEM surrogate to address the high computational cost. Finally, Section 6 provides a discussion of the limitations of the modeling framework along with future prospects, and Section 7 presents our summary and conclusions.

## 2 Methods

### 2.1 Modeling Framework

An overview of the computational and experimental tools utilized in our work is depicted in Figure 1. DEM simulations and experimental results are utilized in this work to explore the efficiency of the milling operation. Material-specific parameters that are used as inputs to the DEM model are estimated by adjusting their values to match experimentally observed velocities and collision frequencies. The operating conditions that contribute to grinding efficiency and total conversion are investigated to link DEM simulation outputs to reaction kinetics. To accomplish these objectives, our multiscale framework includes the (a) development of a high-fidelity DEM that replicates the kinematics of the grinding bodies inside the laboratory-scale reactor; (b) calibration of the material parameters based on high-throughput video experimental data and CV tools; (c) exploration of the DEM simulation to identify phenomena most critical to the mechanochemical process; (d) development of correlations that link the DEM simulations and quality attributes of the final product based on tunable process parameters; and (e) development of a surrogate –DEM framework to reduce the computational cost of DEM simulations. Details of the DEM model and the calibration approach are discussed in Sections 2.2 and 2.3, respectively, followed by the kinetic correlations and the reduced-order model in Sections 4 and 5. All of these steps combined form the bases for the multiscale modeling approach that is used to describe the depolymerization of PET waste in ball-mill reactors.

### 2.2 Discrete Element Method Model Development

A DEM simulation is developed to model the interactions of discrete elements using contact laws. Normal and tangential forces acting on each discrete matter as a result of interactions with other moving bodies and the unit geometry are evaluated, which, in turn, dictate the particle motion. The equations of motion are solved for all entities at each time step. User-defined particle properties including particle sizes, shapes, and materials are selected at the beginning of each simulation along with an appropriate contact model. The Hertz-Mindlin contact model is applied in this work, as it is most appropriate for noncohesive spherical shapes and has been extensively utilized in similar applications in past literature.^69−71^ The time integration for this model is set at 5 × 10^–7^ s. Throughout this work, DEM simulations are run for 0.5 s real-time after steady-state conditions have been reached. In terms of CPU time, this required a total of 5 min for each DEM simulation on a computer with an Intel(R) Core(TM) i9-12900, 3.20 GHz, x-64-based processor and 32 GB of RAM. Additional information on the physics and assumptions involved in contact models can be found in the work of Cundall and Strack.^49^ Detailed description of the equations surrounding DEM calculations can be found in the work of Bhalode and Ierapetritou.^52^ All DEM simulations are performed in EDEM (EDEM solutions, 2021.1) commercial software that incorporates all of the related equations of motion.

#### 2.2.1 Ball-Mill Geometry and Contact Model Parameters

**Figure 2:**
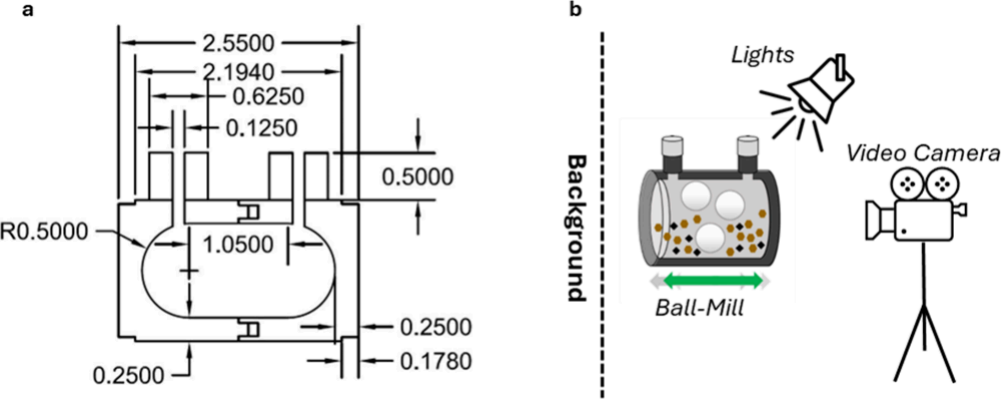
(a) Detailed dimensions in inches of the laboratory-scale ball-mill vessel. (b) Experimental apparatus.

SolidWorks 15.1 is used to create the 3D representation of the reactor geometry that consists of one cylindrical vessel of 25 mL total volume, as per the dimensions of the laboratory-scale ball mill used in the experimental setup (Retch MM400 mill), as shown in Figure 2. The experimental milling system and the DEM digital twin are shown in Figure 4. At the beginning of each computational experiment, the shaking frequency, number of balls and their sizes were fixed, and the grinding media (balls) were generated.

Suitable material and contact model parameter inputs are necessary for an accurate representation of the milling process. The stainless-steel grinding balls are modeled using known steel properties as inputs to the DEM ball-mill model (density 7800 kg/m^3^, Poisson’s ratio 0.3, and Young’s modulus of 210 GPa).^69^ The properties of poly(methyl methacrylate) (PMMA), construction material of the ball-mill vessel, are taken from the work of Falke et al.^72^ (density 1180 kg/m^3^, Poisson’s ratio 0.4, and Young’s modulus of 3.3 GPa). The interaction parameters between the wall and the grinding bodies, coefficient of restitution, and the friction coefficients (static and rolling) were varied iteratively to match the velocity and number of collisions between the simulation and motion-tracking experiments. More details about the estimation of the parameters are provided in Section 3.

### 2.3 Mechanochemical Reactions

**Figure 3:**
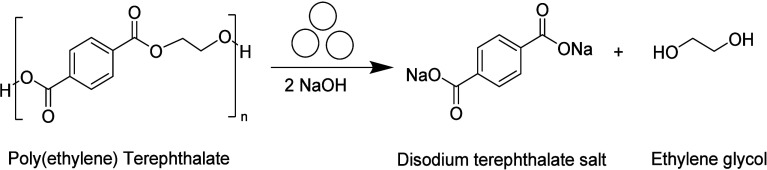
Alkaline hydrolysis of poly(ethylene terephthalate) through ball milling

Poly(ethylene terephthalate) (PET) powder was milled in a Retsch MM400 ball-mill reactor with 2.1 mol equiv of sodium hydroxide (NaOH) relative to terephthalic-acid ethylene glycol repeat units, following the reaction scheme illustrated in Figure 3. The experimental procedure including the materials can be found in Tricker et al.^3^ and is also detailed in the Supporting Information (SI). The resulting products of the mechanochemical hydrolysis reaction are ethylene glycol (EG) and disodium terephthalate (Na_2_TPA). The latter was used to characterize monomer yields using high-performance liquid chromatography (HPLC) according to the procedure detailed in our prior experimental study.^3^ In summary, depolymerization kinetics were explored by investigating the effect of the operating frequencies and ball mass on the achieved conversion. All of the experiments were performed in a 25 mL stainless-steel vessel using stainless-steel grinding balls with diameters of 20 mm at frequencies ranging from 25 to 30 Hz.

The monomer yield was observed to progress linearly until reaching an inflection point of sharp increase in yields, indicating the transformation of the PET/NaOH powder into a homogeneous phase (characterized by the formation of wax). Further experimental investigation of the depolymerization kinetics is outside the scope of this study, while in-depth discussion of the experimental setup and kinetics have been reported in our previous study.^3^ Additional information on the experimental methods can also be found in the SI. The experimental monomer yields obtained from our previous work are used in this work to develop correlations that link the DEM model with the depolymerization yields in Section 4. The achieved yields are depicted in Tables S2–S4.

### 2.4 Design of Ball Milling Experiments for the Calibration of the DEM Model Parameters

A transparent PMMA milling vessel with an interior shape and volume identical to those used in the depolymerization experiments was manufactured and used in motion-tracking experiments without reactants (PET) present, thus neglecting the PET particle-to-particle interactions. This hypothesis has been employed in previous DEM literature,^40,73^ and it is based on the assumption that the collisions between grinding media are far more significant than the collisions between the powder particles (e.g., due to the high relevant mass difference between the two entities). Specifically, the movement of the ball(s) and their average velocities are assumed to not be affected by the presence of powder particles; thus, the kinetic energy and the collision frequency between the ball(s) and walls are not affected. This is important as this allows us to use simulations without powder particles to extract the model outputs used in the proposed model discussed in Section 4. To support this hypothesis, experiments with and without powder were performed and recorded, which validated the assumption that the presence of powder particles (at the specific fill levels of experimentation) does not significantly impact the path of the ball. Video files from experiments including powder can be found in the SI.

The mill’s operation was filmed on the experimental apparatus depicted in Figure 2b using a Chronos 1.4 High Speed camera (2134 fps). A total of eight milling experiments were performed, and the results were filmed. Stainless-steel balls with *d* = 20.6 and 17.5 mm were used in the motion experiments, with the milling frequency varied between 22.5 and 30 Hz. These operating settings were specifically chosen to allow a direct comparison between the observations from the motion experiments and the PET depolymerization yields achieved in our previous experimental study.^3^ The completed list of milling runs is highlighted in Table 1. Video files of the recordings can be accessed in the SI.

**Table 1:** Operating Settings of Recorded Video Experiments

frequency (Hz)	number of balls	diameter (mm)
30, 27.5, 25, 22.5	1	20.6
30, 27.5, 25, 22.5	1	17.5

### 2.5 Object Detection and Tracking Algorithm

The advancements in both optical imaging and machine learning (ML) in the past decade enabled detailed process design and optimization of complex systems with data and measurements that were inaccessible before.^74,75^ CV is a technology suitable for the acquisition, processing, and analysis of visual inputs (e.g., digital images/videos) and, therefore, an integral aspect of automation and calibration for a variety of experimental and computational applications. A simple algorithmic approach was implemented to analyze the movement of objects (grinding balls) between adjacent frames and evaluate the velocities of the balls. The data were then utilized to fine-tune the material parameters of the DEM model and replicate the motion experiments.

The OpenCV (Open-Source Computer Vision) and Numpy libraries^76−78^ in Python are used for object tracking and image processing. The MOSSE tracker (Minimum Output Sum of Squared Error) is utilized to identify the moving ball(s). MOSSE is known to be very robust, especially for identification of high-speed objects (such as the milling balls) or changes in lighting and scale.^76,77^

**Figure 4:**
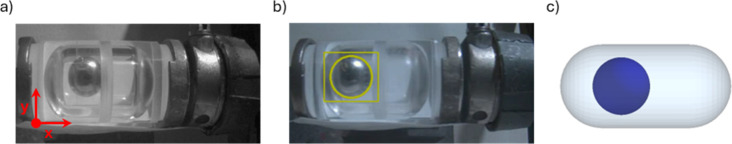
(a) Experimental setup includes a vibratory 25 mL reactor and one stainless-steel ball of varying diameters. (b) The OpenCV computer vision python library is used to track each ball as it moves inside the milling vessel. (c) Replication of the experimental setup using the DEM software.

The positions of the balls in the *x*- and *y*-directions were recorded. The influence of the *z*-direction on the total velocity is assumed to be insignificant due to the relatively small reactor volume. Once the coordinates of the ball in space and time were identified, the velocity was calculated as the change in position between two consecutive frames using eqs 1–3. In addition, after a collision occurs, the ball changes direction; thus, the number of collisions is identified as the number of times the sign of the velocity vector changes. The ball diameter was used as the standard to measure the distance in the videos, and the time step was set as the video frame rate. Figure 4a,b illustrates the video footage for one milling ball and its detection from the CV algorithm.
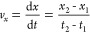
1
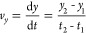
2
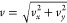
3

## 3 Results

### 3.1 Discrete Element Method Model Calibration

#### 3.1.1 High-Speed Video Analysis

**Figure 5:**
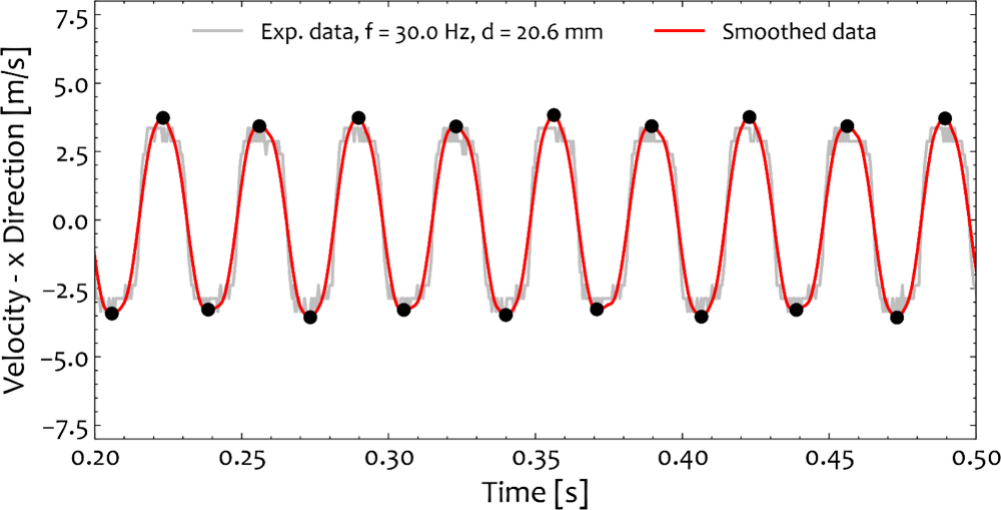
A typical segment of the *x*-direction of the velocity vector for the case of one stainless-steel ball with a diameter equal to 20.6 mm 30 Hz milling frequency as evaluated from the recorded experiment and the object detection algorithm. The gray line illustrates the raw experimental data, while the red line denotes the smoothed velocity data. The collision events are denoted by black circles.

To study the milling process, the collected video experiments are analyzed. Figure 4 a,b illustrates frames from the raw video data and the detection of the balls using the OpenCV computer vision algorithm (Raw video data can be found in the SI). The horizontal (*x*) and vertical (*y*) positions of the grinding ball are tracked and extracted from each recorded experiment. The Savitzky–Golay filter was then utilized to smooth the collected velocity data and eliminate any experimental noise, as shown in Figure 5. Figure 5 displays a representative section of the ball velocity in the *x*-direction (_ν*x*-ball_), as well as the collision events for a milling frequency of 30 Hz and *d*_ball_ = 20.6 mm. Two end-on collisions in the *x*-direction occurred per milling cycle, with ν_*x*-ball_ remaining relatively constant. Additionally, the velocity in the *y*-direction is approximately one order of magnitude lower, confirming this observation. Velocity and collision time evolutions were extracted from the recorded experiments shown in Table 1 and were utilized to calibrate the DEM material and contact parameters.

#### 3.1.2 Calibration of the DEM Model

The DEM simulations were executed with the initial set of material parameters as inputs. Next, the comparison metrics (average velocities and collision events) were extracted at every time step under the same operating conditions as those for the recorded milling experiments. The material parameters were adjusted guided by the sensitivity analysis results (discussed in Section 3.2) to match the experimentally observed features. Finally, the material parameters were considered to be calibrated when the relative difference of the comparison metrics was insignificant.

Experiments performed for one stainless-steel ball with diameters of 17.5 and 20.6 mm and milling frequency ranging from 22.5 to 30 Hz are chosen as the validation set. As an initial estimate, the material and contact parameters for steel-PMMA interactions were chosen based on the work of Falke et al.,^72^ where properties of materials similar to the ones used in our experimental setup were evaluated. Lower and upper bounds were set in such a way that they restrict the available search space based on the physical meaning of the interaction parameters. A sensitivity analysis was initially performed to guide the parameter estimation process and identify a combination of parameters that correspond to velocities and collision frequencies close to the ones extracted from the experimental data and the CV algorithm.

**Figure 6:**
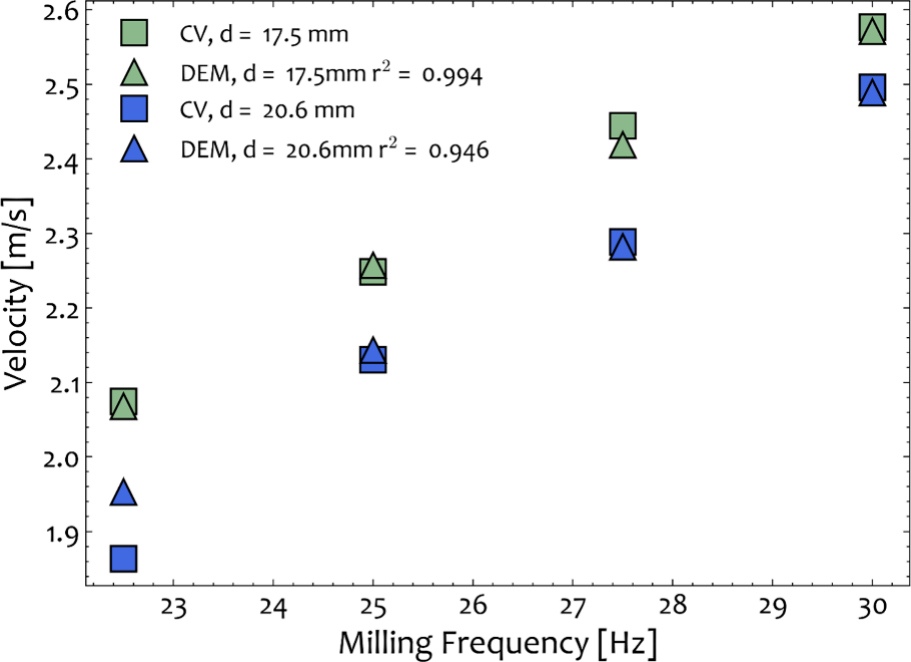
Average velocities measured from the recorded experiments (squares) and the DEM simulations (triangles) for *d*_ball_ = 17.5 mm (green) and *d*_ball_ = 20.6 mm (blue) for the identified set of material parameters. The velocities are compared for different vibration frequencies (22.5–30 Hz).

**Figure 7:**
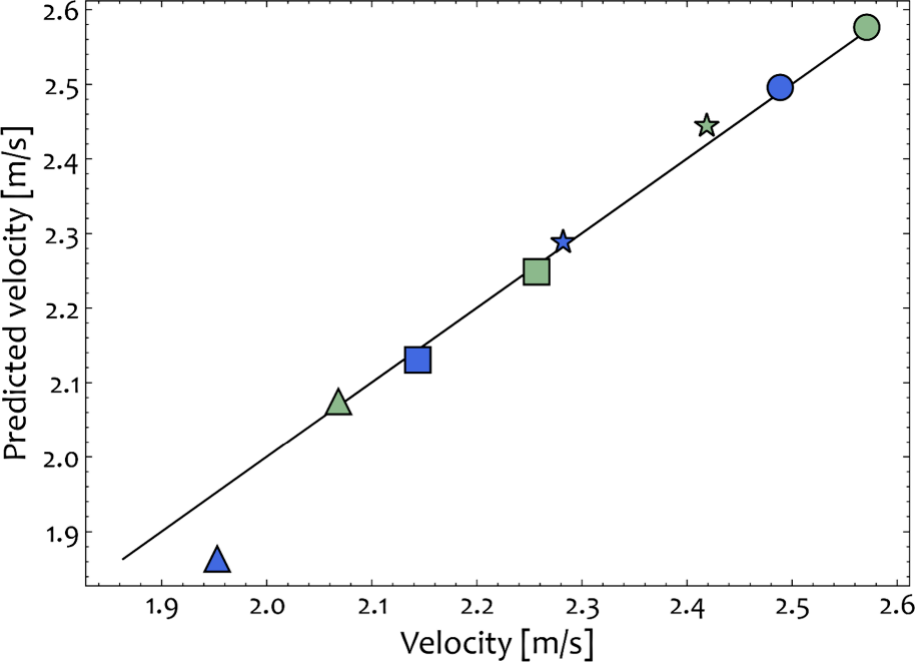
Comparison of predicted and measured velocity from the recorded experiments and the DEM simulations for *d*_ball_ = 17.5 mm (green) and *d*_ball_ = 20.6 mm (blue). Triangle, square, star, and circle symbols denote the frequency of operation that was set at 22.5 25, 27.5, and 30 Hz, respectively.

The final set of parameters and their respective bounds for the two validation cases tested (*d* = 17.5 mm and *d* = 20.6 mm) are presented in Table 2. For this set, the average ball velocity is compared in Figures 6 and 7 for the validation cases, which reveals that the values are in very good agreement for the different operating conditions tested and can thus validate the model (Table 3). The reader is referred to Figure S1 for a comparison of the velocity trajectory between the experimental and DEM data. A coefficient of restitution (*C*_R_), a static friction coefficient (SF), and a rolling friction coefficient (RF) equal to 0.11, 0.7, and 0.5, respectively, were identified. Known material parameters such as densities, Young moduli, and the Poisson’s ratio were not varied.

**Table 2:** DEM Material Parameters, and their Respective Bounds in the Sensitivity Analysis Study

	symbol	value	bounds
density, ball (kg/m^3^)	ρ_ball_	7800	constant
density, wall (kg/m^3^)	ρ_wall_	1180	constant
Young modulus, ball (GPA)	*E*_ball_	210	constant
Young modulus, wall (GPA)	*E*_wall_	3.3	constant
Poisson ratio, ball [−]	ν_ball_	0.3	constant
Poisson ratio, wall [−]	ν_wall_	0.4	constant
coefficient of restitution, ball–wall [−]	C_R_	0.11	0.1–0.7
static friction coefficient, ball–wall [−]	SFμ_s,b–w_	0.7	0.3–0.7
rolling friction coefficient, ball–wall [−]	RFμ_r,b–w_	0.5	0.1–0.5
time step [s]	Δ*t*	5 × 10^–7^	
simulation time [s]	*t*	0.5	

Once the DEM parameters are estimated, the simulation can be executed under different conditions and the results can be used to further analyze the ball milling system. In the future, if powder particles or other entities are introduced within the DEM simulation, then new parameters (e.g., coefficient of restitution, static, and rolling friction) will be required to describe the interactions between PET particles and balls/walls. The parameters that are identified in this work that describe the interactions between the ball(s) and walls will not be affected.

**Table 3:** Average Velocities and Collision Frequency as Calculated from the Recorded Experiments and the DEM Simulation

milling frequency (Hz)	ball diameter (mm)	average velocity (m/s)	average velocity DEM (m/s)	collision frequency (1/s)
30	17.5	2.57	2.57	60
	20.6	2.50	2.49	60
27.5	17.5	2.44	2.42	55
	20.6	2.29	2.28	55
25	17.5	2.25	2.26	50
	20.6	2.13	2.14	50
22.5	17.5	2.07	2.07	45
	20.6	1.86	1.95	45

### 3.2 Sensitivity Analysis for the Material-Based DEM Parameters

Sensitivity analysis is used to qualitatively and quantitatively analyze the extent of variability in the response of a mathematical formulation to design or operational variables. The main goal of such an analysis is to identify the importance of each material parameter to the model response^69,79^ and determine their effects on complex model formulations.^69,79−81^ Additionally, the issue of solution multiplicity whereby various material parameter values might result in solutions that fit the data may occur, since the corresponding system of equations is underdetermined.^52,60,82^ This is a common challenge in the bulk measurement material parameter identification problem, where there is not one unique solution to the system. In this work, we use a variance-based sensitivity analysis approach to exploit how the material-based parameters affect the overall behavior of the model. Specifically, our objective is to evaluate the impact of calibrated material parameters on the predicted average velocity and identify if multiple combinations of contact parameters can lead to a similar model response. Overall, sensitivity analysis studies map the system response subject to the values of the material parameters and allow for further exploitation of the system and accurate calibration of the parameters.

**Figure 8:**
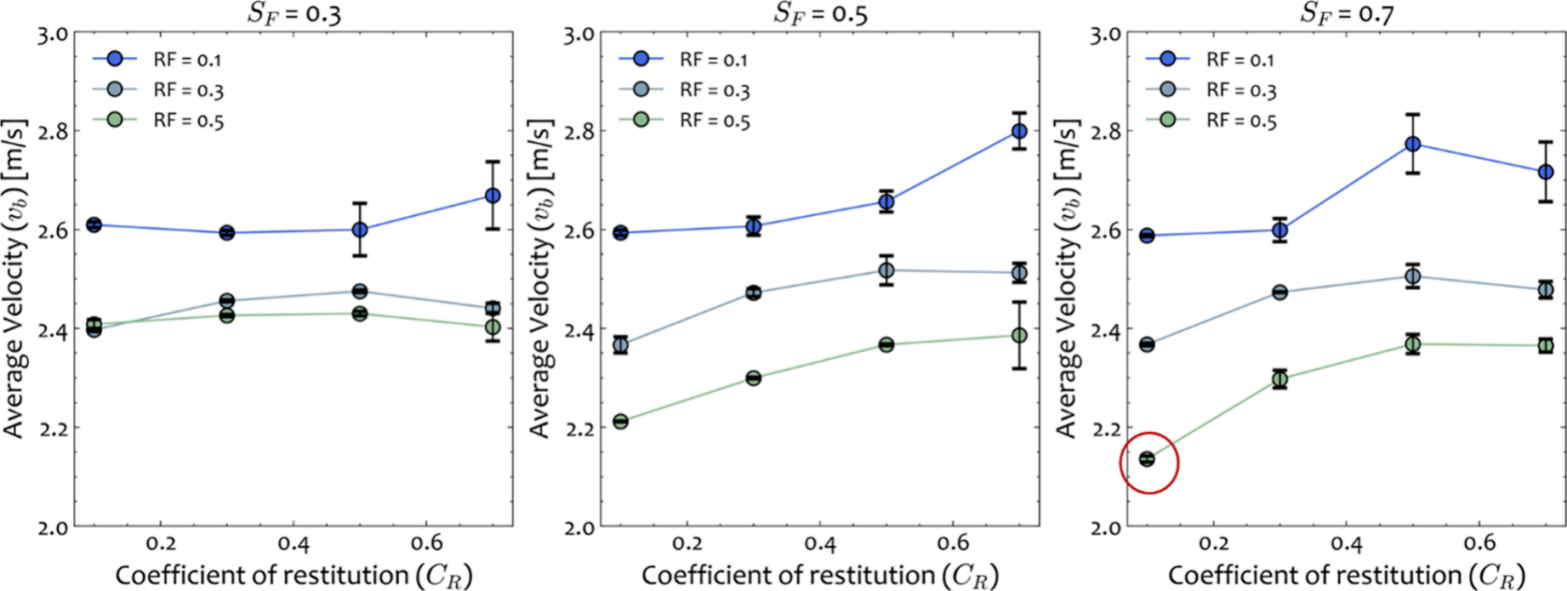
Average velocity values for the case of one stainless-steel 20.6 mm ball at a milling frequency of 25 Hz for different rolling (RF) and static friction (SF) coefficients and coefficient of restitution values (*C*_R_). The red circle indicates the value of the ball’s velocity as extracted from the experimental data for the particular set of operating conditions.

The coefficient of restitution (*C*_R_), rolling friction (RF), and static friction (SF) for stainless-steel and PMMA interactions are changed iteratively within their respective bounds, as shown in Table 2. The coefficient of restitution determines the relative velocity effect of the (in)elasticity of two colliding bodies. Hence, high values of coefficient of restitution will lead to higher velocities and vice versa, as is observed in Figure 8. The coefficient of rolling friction determines the required torque to be applied to an object at rest on a given surface to put into rolling motion, while the coefficient of static friction determines the required normal force to be applied to the same object to begin moving. For very low static friction coefficient values, the grinding ball moves faster indicating that small force and torque requirements are necessary for its movement.

Through this sensitivity analysis, we see that all parameters are important and strongly influence the resulting average velocities. In Figure 8, we report the averages and standard deviations from results of three sets of simulation runs. Replications are performed to quantify the variability of the results caused by the stochasticity of the DEM simulation initialization. It can be seen that the effects of the three material parameters to the final average velocity are nonlinear. In addition, solution multiplicity is observed at some instances for low coefficient of static friction (SF = 0.3) where similar average velocities are identified for different combinations of parameters. The solution multiplicity for the bulk measurement approach is a known issue, and hence, proper upper and lower bound values should be chosen in such a way that parameter values with no physical meaning are excluded from the grid search. For the present case (*v*_avg_ = 2.13 m/s), it was found that the set of parameters that corresponds to the measured velocity is unique and the solution multiplicity is not observed at this material parameter space. All in all, the sensitivity results in this work allowed for the mapping of the system’s response to further guide the simulation runs in the identified regions and identify material parameters that lead to results consistent with the experimentally measured values.

### 3.3 Influence of Milling Parameters on the Kinetic Energy of the Grinding Balls

Once the materials and contact parameters of the DEM model are calibrated, and the kinematics are accurately captured, DEM simulations can be executed to investigate the parameters that influence the mechanochemical process. Specifically, the entities within the reactor system are tracked over simulation time to evaluate the influence of the degrees of freedom (e.g., milling frequency and ball sizes) on the kinetic energy which is known to be important for characterizing mechanochemical reactions.^83,84^ This systematic analysis provides the necessary tools for identifying the optimal operating regime that will lead to higher monomer yields.

**Figure 9:**
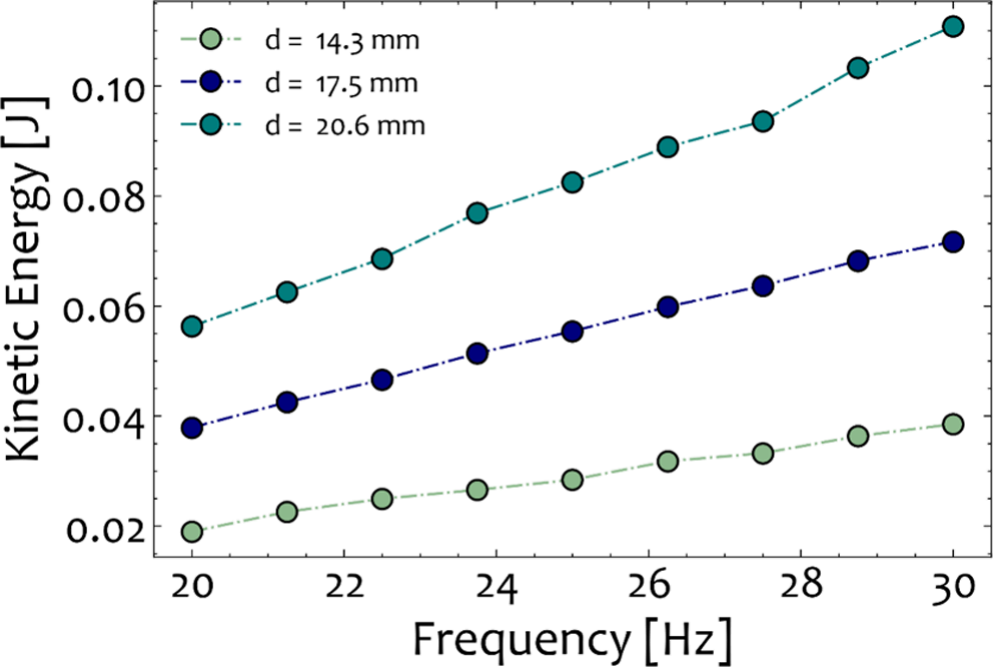
Average kinetic energy of a mill operating with one stainless-steel ball of different sizes operating at different milling frequencies.

Figure 9 illustrates the relationship between the average kinetic energy, the ball sizes, and the milling frequencies. For lower milling frequencies, the evaluated average kinetic energy is relatively small for all the studied combinations of sizes. In our previous experimental study on the depolymerization kinetics within the same mill,^3^ it was shown that for milling frequencies lower than 25 Hz, the extent of the depolymerization reaction is rather small. This implies that the impact intensity of the collisions and the mechanical energy induced are not sufficient to activate the mechanochemical reaction, assuming that the behavior of the mill for the remainder of the reaction time (∼20 min) will follow a similar trend. In contrast, for higher milling frequencies and ball sizes, complete depolymerization was achieved at smaller milling times.^3^ This observation is quantified with the results of the DEM simulations, which indicate that under these conditions, the average kinetic energy of the grinding media is considerably higher. In general, increasing the ball size and the vibration frequency had a positive effect on the kinetic energy (*E*_kin_) during ball milling. This analysis constitutes the basis of the following section, which links the outputs of the DEM simulations to mechanochemical yields.

## 4 Linking DEM Outputs to Mechanochemical Yield

Understanding and quantifying kinetic information on a reaction is an essential step toward predicting the reaction pathway, controlling, and optimizing the reactor, and eventually assessing efficiencies at larger scales. The kinetics of most of the reactions are dictated by their energy descriptor, i.e., temperature or potential in thermochemical and electrochemical reactions, respectively. For the case of mechanochemical reactions, it has been demonstrated that the critical energetic descriptor is the kinetic energy of the moving balls.^17,84^ In other words, the reaction rate is expected to be proportional to the milling intensity, via a kinetic parameter *a*, and consequently to the kinetic energy of the ball and collision frequency. Measuring parameters such as velocities, however, often requires sophisticated experimental setups to record the milling process, while the estimated kinetic parameters will still be dependent on the system specifics. DEM models can computationally estimate the kinetic energy and collision frequencies (i.e., the energy descriptor) for different sets of operating conditions without the need for performing expensive experiments. This will enable the use of the model for the efficient exploration of conditions that would lead to optimal performance. Of course, the exact value of the proportionality parameter *a* is expected to be dependent on the system specifics not captured by DEM in this work, such as the substrate or the fill level.

Linking data from DEM simulations with the achieved yields will further enable exploitation of the reaction kinetics for mechanochemical reactions. To accomplish this goal, we utilize experimental results discussed in our previous experimental study for PET depolymerization,^3^ and results from DEM simulations performed for the same operating settings. These are subsequently used to construct correlations to relate the milling parameters to the depolymerization kinetics. This way, information about the systems’ specifics such as the type or size of grinding media and reactor can be incorporated.

### 4.1 Relationship between the Total Energy and Reaction Yields

**Figure 10:**
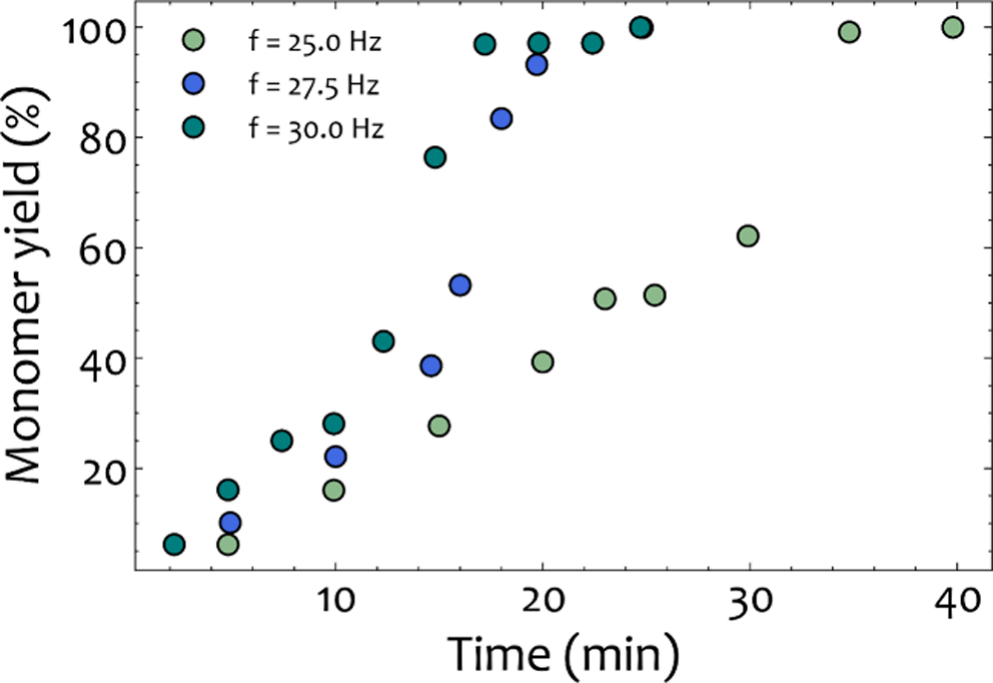
Raw experimental data for the monomer yield as a function of depolymerization time for the case of one stainless-steel 20 mm at 25, 27.5, and 30 Hz.

Figure 10 depicts the progression of depolymerization over time for PET samples (1 g of PET + 0.42 g of NaOH) milled with one stainless-steel ball (*d* = 20 mm) at operating frequencies of 25, 27.5, and 30 Hz. The monomer yield initially follows a linear relationship with respect to the milling time (*t* ≤ 12.5 min) until an inflection point after which the depolymerization rate increases rapidly and complete depolymerization is reached within 17.5–38 min depending on the operating conditions. This inflection point corresponds to the transition of the PET/NaOH powder to a homogeneous waxy phase. Finally, complete depolymerization of PET powder was observed at 20, 25, and 40 min for 30, 27.5, and 25 Hz, respectively. The transition to wax phase is not instantaneous, and for some time, the contents of the reactor are a mix of powder and wax. A comprehensive table with all of the experimental results highlighting the yield and the corresponding phases is provided in Tables S2–S4.

**Figure 11:**
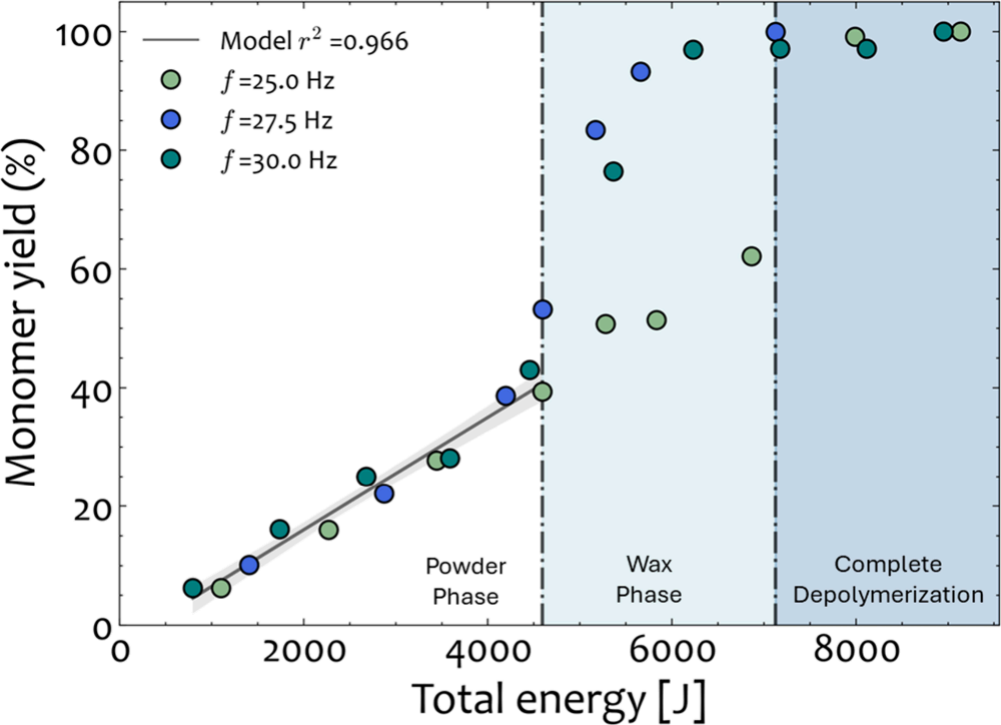
Monomer yield as predicted using the kinetic energy and frequency of collisions from the DEM simulations for the case of one stainless-steel ball with *d* = 20 mm at *f* = 25, 27.5, and 30 Hz. The boundary between the "Powder" and "Wax" is drawn at 4593 J, while between the "Wax" and "Complete Depolymerization" phases at 7126 J.

DEM simulations are executed, and simulation results are extracted for the operating settings for which monomer yield data are available. To study energetics, we extract from each simulation run (*f* = 25, 27.5, and 30 Hz) the kinetic energy (as a function of the average velocity) and the frequency of collisions per unit time. In Figure 11, the correlation between the cumulative kinetic energy supplied to the milling system over time and the observed monomer yield is depicted. When plotted against milling time, the monomer yield followed different trends for the three operating settings (*f* = 30 27.5, and 25 Hz), as shown in Figure 10; however, a distinct, unified trend emerges when plotted against the energetic descriptor of the reaction (Figure 11).

In the initial reaction regime, termed the “powder phase”, linear progression of yields with milling intensity is observed across all operating conditions. Samples taken from the ball mill during this regime were uniformly in the powder phase. The transition out of the powder phase occurs after a cumulative energy dose of *approximately* 4600 J across all three operating conditions. Subsequently, an intermediate phase, termed the “wax phase”, occurs where the reactant material consists of a hybrid mixture of powder and wax. Finally, the system transitions into the homogeneous wax phase regime, indicated by the “complete depolymerization”. This final regime is achieved once the cumulative energy input exceeds 7100 J. Beyond that point, additional energy input no longer influences the depolymerization of the PET, as Na_2_TPA monomers have already been formed.

These observations facilitate the formulation of a linear expression for the powder regime that will establish a connection between the yield with the DEM simulations within that region. This will enable the prediction of the point of the phase transition and conversions up to approximately 40%. After progression to the wax and complete depolymerization regimes, the relationship between energy and yields is not linear and the data exhibit greater spread. The phase transition event occurs suddenly, and conversion rapidly accelerates toward complete depolymerization afterward. It is very difficult to predict precisely when the onset of the phase transition occurs due to high sensitivity to the prior history of the reactant mixture up to that point. There is, however, a clear boundary between partial and full depolymerization that can be drawn. A model that would predict the entire trajectory from the powder to complete depolymerization phase could be trained using nonlinear regression. However, because of the limited data and additional uncertainty introduced at the transition between the powder and wax phases, it is hard to propose such correlations that would be interpretable and validated. The different regions are colored in these two regimes to broadly estimate the range of possible conversions for energy inputs above ∼4600 J. All in all, if the cumulative energy is higher than ∼7100 J, full depolymerization can be achieved.

The linear function that describes the powder phase is calibrated using the available experimental data for the three operating conditions (*f* = 25, 27.5, and 30 Hz). Since the precise phase transition point is unknown, several linear functions are evaluated and the best set of equations is selected by minimizing the least-squares values. The regression line is accompanied by a shaded region signifying a 95% confidence interval, indicating the range where the actual regression line lies. The kinetic expression that was identified is shown in eq 4 while the kinetic parameters are reported in Table 4.
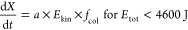
4where *E*_kin_ [J] is the average kinetic energy, *f*_col_ [1/s] is the collision frequency, and *a* and *b* (intercept) are fitted to the experimental data. *X* represents the yield, as defined in eq 5. The lumped parameter *a* captures the importance of feedstock properties. This way, the high-fidelity DEM data are combined with the experimental results to predict the monomer yield. The effects of the ball size, geometry, and materials are all accounted for by the DEM model. Using this lumped parameter approach, the use of the DEM model enables prediction for other operating settings (e.g., ball sizes, frequency, and geometries).
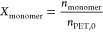
5

**Table 4:** Kinetic Parameters Identified for eq 4

	kinetic parameter (slope), *a* [mol_monomer_/mol_PET,0_ kJ^–1^]	intercept, *b* [mol_monomer_/mol_PET,0_]
powder phase regime	9.49 ± 1.00	–3.03 ± 1.70

Table 4 reports the identified kinetic parameters that characterize the link between the energy descriptor and the yield. The nonzero *y*-intercept (*b*) indicates that there is a short “induction” period at the start of milling where solid–solid mixing and particle size reduction dominate before depolymerization takes over. The predicted and experimental values are in satisfactory agreement (*r*^2^ = 0.966), indicating that once trained, the equations showcased in this section can be utilized to estimate conversions using only simulation results.

The advantage of the proposed method is that after training, it does not require any experimental data as input for predicting the extent of depolymerization up until the phase transition and will only utilize results from the DEM simulations as well as broadly estimate the range of complete depolymerization. In addition, the model proposed here is expected to provide good predictions within the range of the training data. In other words, if any of the parameters that were kept constant during our training procedure changes such as the amount of PET milled or the fill level, the regression parameters of eq 4 can be optimized using new experimental data; however, the general behavior of the reaction as a function of energetics should remain the same. Finally, it is acknowledged that once the phase transition occurs from powder to wax, the results of the DEM simulation might not be as accurate. This is because parts of wax are likely to stick to the ball surface and, therefore, might explain why there is no clear trend between energy dose and yield in the wax and complete depolymerization regimes.

## 5 Reduced-Order Model for the DEM Simulation

The validated DEM model, even with a few grinding entities, requires setup and simulations with specialized DEM software. Once validated, the DEM model can be used to generate enough data a priori to fit a fast surrogate model that accurately captures the important input–output correlations required for future tasks (i.e., process integration and design). Surrogate models are often employed to describe systems of equations that are expensive to solve and thus reduced through efficient surrogates to lower cost and faster models.^67,85^ These reduced-order representations can be later embedded within a process flowsheet simulation or other models, suitable for process optimization, control applications or further system analysis.^67^

### 5.1 Methodology for the Construction of the Reduced-Order Model

In the current work, mechanistic input–output data obtained from DEM simulations are used to fit low-cost regression models (surrogates) that represent the mechanistic data. The outputs predicted by these surrogates are the inputs to the DEM-mechanochemical reaction model (DEM-MC) developed in Section 4. The inputs selected are the milling frequency and ball-to-reactor volume ratio (BVR), which are inputs that are scalable. Our hypothesis is that we can identify a simple regression model to capture the important links between these critical process inputs and DEM outputs.

**Figure 12:**
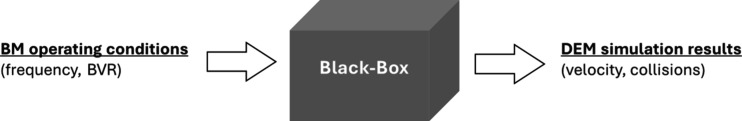
Surrogate model for DEM ball-mill simulation.

Toward this effort, we used rigorous training and compared a variety of regressors, including linear regression, random forests, support vector regression, and neural networks. Results are presented here for the two top models that were found to balance accuracy and simplicity, namely, linear and random forest (RF) regression. Both surrogate techniques have been utilized in a variety of applications in the literature to reduce the cost of expensive simulations. A simple schematic of the surrogate model–mechanochemical reaction model (SM-MC) training is shown in Figure 12, where the ball-mill operating conditions, namely, the milling frequency and the ball-to-reactor volume ratio (BVR), are used as inputs while the output of the model are the DEM simulation results used for predicting the monomer yield. The BVR ratio is chosen such that the SM-MC model can be used to estimate the conversion in reactors other than the one used in the experimental work.^3^

The collision frequency and the average kinetic energy of the ball (outputs of the DEM simulation) are used as inputs to the MC model (e.g., eq 4). Subsequently, we built the reduced-order model to predict those two sets of variables given the ball-mill operating settings: the frequency and the BVR parameter. Linear regression and random forest models are built on data obtained from 27 DEM simulation runs. The data correspond to average velocity and collision frequency for operating frequencies ranging from 20 to 30 Hz (equally spaced at a step of nine increments) and ball radii equal to 14.3, 17.5, and 20 mm, respectively. Both models are trained using the sklearn library in Python.^86,87^ Mathematically, the regression problem can be presented as follows:
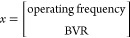
6
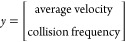
7
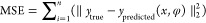
8
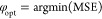
9

In the context of this problem, *x* and *y* stand for the input and output vector of the surrogate model, respectively. The mean squared error (MSE) loss function is utilized to quantify the disparity between predictions and actual values. The solution of the mathematical problem results in the optimum set of hyperparameters (φ_opt_) and the corresponding *y*_predicted_ vector. The optimal hyperparameters of the random forest model are determined through a comprehensive grid search over a wide range of values to ensure that the parameter space is thoroughly explored.

To fit the regression models, we split the data set into training and testing sets. 70% of the data are used for training to find the optimal model parameters. The remaining 30% of the data are used for testing purposes and for monitoring the generalization performance. To monitor the accuracy of the resulting models, we compare the R^2^ scores, the percentage errors and the parity plots.

### 5.2 Surrogate Model Results

**Figure 13:**
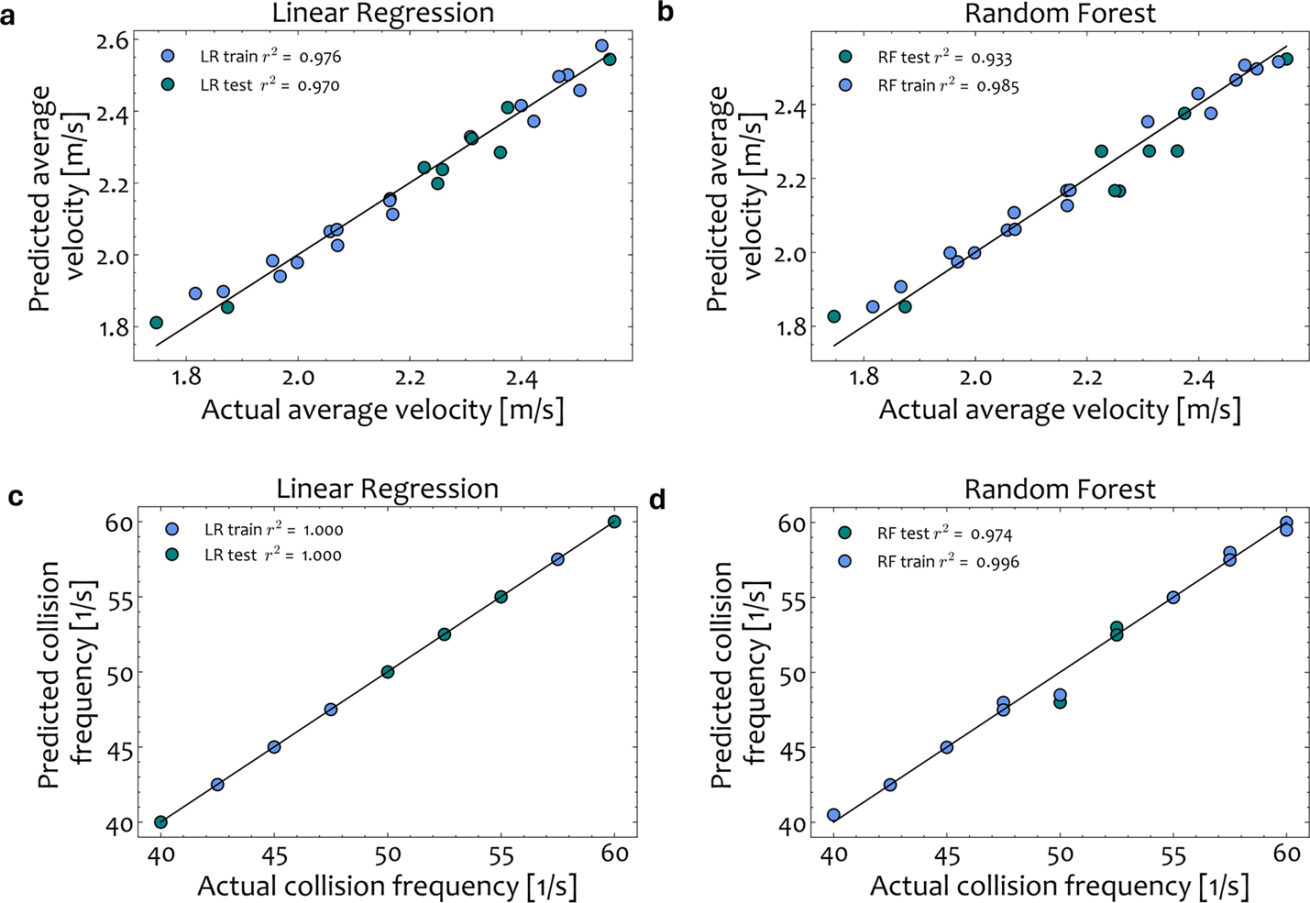
Parity plots for the average velocities and collision frequency reduced-order models from linear regression (a, c) and random forest (b, d) for the training and test data sets.

The performance of the trained surrogate model (*y*_predicted_) is first compared with the results derived from the high-fidelity DEM simulation (*y*_true_) for the three validation cases. The parity plots for the average velocities and collision frequencies are depicted in Figure 13, while Table 5 lists the error metrics. The percentage errors from the surrogate approximations as depicted in Table 5 and Figure 13 are less than 2% for the velocity predictions and less than 4% for the collision prediction for both surrogates, with linear regression showing better performance.

**Table 5:** Percentage Errors for Average Velocity and Collision Frequency Surrogate Models for the Three Validation Cases with Respect to the DEM-Computed Values

	**average velocity percentage error (%)**		**collision frequency percentage error (%)**	
	LR	RF	LR	RF
case study 1 (*r* = 10 mm and *f* = 25 Hz)	0.38%	1.76%	0.26%	3.26%
case study 2 (*r* = 10 mm and *f* = 27.5 Hz)	0.88%	1.96%	0.74%	0.74%
case study 3 (*r* = 10 mm and *f* = 30 Hz)	0.76%	0.98%	3.36%	3.36%

**Figure 14:**
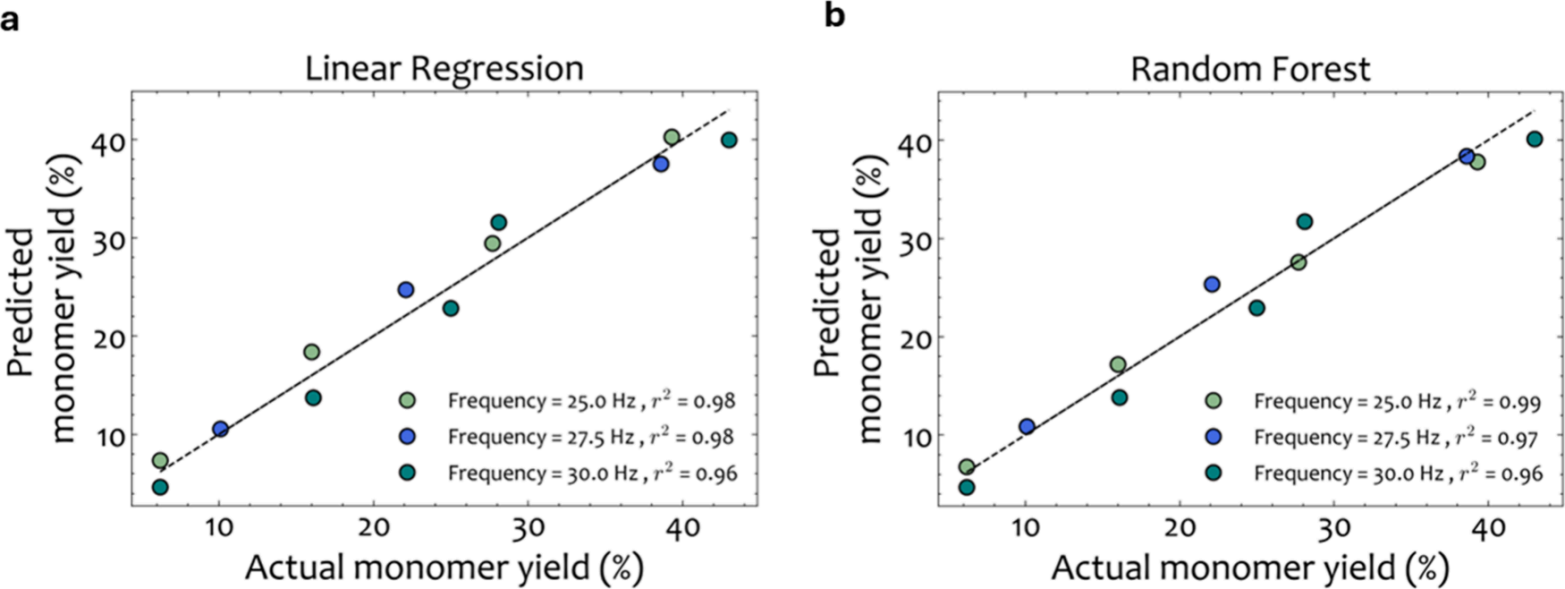
(a) LR-MC and (b) RF-MC parity plots comparing the actual and the predicted monomer yield for *f* = 25, 27.5, and 30 Hz.

To determine the acceptable error threshold, however, it is crucial to compare the hybrid framework performance (SM-MC) with the experimental results to evaluate how much the chosen surrogate model affects the prediction of the monomer yield and not just the DEM outputs. Therefore, the model prediction of the DEM-MC and SM-MC approaches should be compared through the predicted yield to evaluate the information loss and the prediction accuracy of the reduced version in comparison with the high-fidelity approach. Table 6 reports the absolute errors between the predictions of the DEM-MC and SM-MC approaches and the experimental data for all operating settings. Additionally, to highlight the prediction accuracy of the LR-MC and RF-MC surrogate models, the parity plots comparing the actual (experimental) and predicted yields (SM-MC) are shown in Figure 14 a and b, respectively. These findings suggest that both surrogates can effectively predict the resulting MC yield for the case of PET waste in a fast manner.

**Table 6:** Absolute Errors for the DEM-MC, LR-MC, and RF-MC Models for Case Study 1, *r* = 10 mm and *f* = 25 Hz, Case Study 2, *r* = 10 mm and *f* = 27.5 Hz, and Case Study 3, *r* = 10 mm and *f* = 30 Hz

	Na_2_TPA yield absolute error (|X_pred_ (%) – X_exp_ (%)|)		
	DEM	LR	RF
case study 1 ( *r* = 10 mm and *f* = 25 Hz)			
maximum	2.55	2.39	1.51
minimum	1.23	0.94	0.12
case study 2 ( *r* = 10 mm and *f* = 27.5 Hz)			
maximum	2.14	2.63	3.23
minimum	0.24	0.47	0.23
case study 3 ( *r* = 10 mm and *f* = 30 Hz)			
maximum	3.71	3.46	3.61
minimum	1.66	1.55	1.51

While both surrogate approaches show similar capabilities regarding prediction accuracy, it is important to consider other factors that are associated with the development of the model and its implementation. For example, in comparison to a random forest model, a linear regression model is easier to set up and physically interpret. Taking into account the prediction accuracy presented in Tables 5 and 6, as well as the complexity of the resulting surrogate model, we conclude that the LR-MC modeling approach is favorable in comparison to the more complex random forest approach. This case study demonstrates that the SM-MC approach can achieve comparable results with the DEM-MC approach while offering significant advantages in terms of computational efficiency. The SM-MC model can generate results within seconds, whereas the DEM-MC approach might take more time to run. The required run time will significantly increase when studying the scaling-up procedure of the ball milling depolymerization process, a necessary step toward establishing mechanochemical processing of plastic waste. All in all, the SM-MC approach offers a significant advantage of being able to predict the behavior of the system without the need for running expensive DEM computations at unseen conditions.

## 6 Discussion

Throughout this work, we illustrated the use of DEM models to evaluate the energetics associated with the use of ball-mill reactors for depolymerization purposes and the significance of the cumulative energy dose to the resulting yield. To achieve this objective, we trained a hybrid DEM-MC model, which establishes a connection between the high-fidelity DEM model and experimental data through a lumped parameter, parameter *a*. Using lumped parameter *a*, we capture the importance of feedstock properties. This computational framework can be used to predict the conversion without the need for performing additional expensive experimentation. One limitation of the equations presented in this article is that they cannot be directly used for extrapolation and are valid only within the ranges of the available experimental data (e.g., PET and NaOH quantities, fill-level). Instead, a framework employing models of varying fidelity to describe mechanochemical depolymerization is showcased. Once the DEM-MC hybrid framework is trained with appropriate data that describe other types of feedstocks or fill levels, it can be employed to elucidate results that can be generalized to other types of ball mills to obtain the same performance.

One additional limitation is that the breakage of PET powder is assumed to have no influence on the total energy or the calculated yield. While this assumption is reasonable for the type of raw materials and fill levels explored in this work, the incorporation of granular materials or irregular plastic shapes that resemble actual waste materials into the DEM simulations can provide more insight into how the PET particles break and are converted to monomers. This will make the model more reliable by evaluating the impact of the shape and material properties on the process output, and will further enable the holistic understanding of mechanochemical depolymerization for plastic recycling, and the development of generalizable models to predict monomer yields for different types of feedstocks.

One significant application of the proposed modeling framework is the simulation and quantification of energetics and efficiency of the operation of industrial-scale ball-mill reactors. A DEM simulation can be virtually constructed and, based on the simulation results, estimate the residence time required to complete depolymerization or calculate the costs associated with the operation of a ball-mill reactor to attain a specific yield. In our prior studies,^88,89^ we established a data-driven correlation to connect the yields achieved within the reactor with the ball-to-powder (BPR) ratio and conducted DEM simulations for industrial-size reactors to calculate the operating expenses. By integrating the DEM-MC framework introduced in this study for an industrial-size reactor, it becomes feasible to predict the operating costs to achieve a certain degree of depolymerization. Such investigations can be very significant, as one of the primary concerns regarding the potential of mechanochemical methods for waste processing is the high operating costs tied to the operation of industrial-size ball-mill vessels. A valuable extension of this would be to investigate potential strategies to optimize operating costs in a manner that would ensure complete depolymerization. One of the anticipated challenges when moving from the lab to industrial scales is the uncertainty introduced to the computational results due to changes in reactor geometries (e.g., vibratory versus rotating reactor). Simulation tractability challenges will arise in scale-up studies if it becomes important to simulate grinding media and raw materials (e.g., powders, flakes, or films). Furthermore, challenges may arise by the presence of a significant amount of wax at industrial-scale reactors, even though this does not impact the internal path of the ball at the lab scale.

Additionally, there is a huge body of literature that connects DEM models with population balance models (PBMs), which have been used to describe the operation of particulate processes in pharmaceuticals and mineral processing.^69,71,90,91^ There is significant value in developing models that can capture the molecular weight distribution (MWD) during depolymerization under varying operating conditions (e.g., shaking frequency, number of steel balls, catalyst, carrier gas, geometry), to enable design of the reactor and subsequent separation systems. A fully mechanistic model would be ideal, but it remains infeasible due to the inherent complexity of the ball milling process and the large variability in operating conditions between systems, including mill geometry and selection of catalyst. This kind of work would lead to the development of a physics-based PBM model that can predict the evolution of MWD over time in relation to the energy doses extracted from the mechanistic DEM model.

Moving along, an industrial-scale ball-mill reactor model can be incorporated within a flowsheet simulator to enable the process flowsheet design and optimization. However, because process simulators require fast estimates to run, the connection with high-fidelity DEMs is infeasible. In this case, the surrogate approximations developed in this work can be more easily integrated within flowsheet software. Such studies may allow the investigation of potential economic and processing trade-offs between the operation of the ball-mill unit with the downstream separation/purification steps. We anticipate that process-level analyses will be critical for comparing this novel technology with alternative recycling routes and aid policymakers and industry stakeholders in making informed decisions regarding its overall potential.

## 7 Conclusions

This article presented an integrated approach for modeling mechanochemical depolymerization in ball mills using DEM models, linking operating conditions to reaction yields. The DEM material parameters were calibrated to best represent the kinematics of the milling system. The simulation was validated via a grid search and a sensitivity analysis study. Subsequently, the validated model was utilized to explore the energetics of the milling system and investigate the influence of the operating parameters on the achieved depolymerization yield. It was found that for combinations of small grinding ball sizes and low milling frequencies, the kinetic energy is low, which hinders the depolymerization reaction. In contrast, larger sizes and milling frequencies result in higher kinetic energies and increased monomer yields.

A mathematical model was then formulated to link the high-fidelity DEM simulation outputs (velocity profiles and collision frequency) to the attained depolymerization yields. Three distinct regimes of operation (e.g., the powder, the wax, and the complete depolymerization phases) were defined based on the cumulative energy supplied. A linear relationship was established to predict the progression of yield in the powder phase regime and estimate the onset of the phase transition. The derived equations can be effectively applied to predict the monomer yield at unseen operating conditions using the DEM simulation results as inputs without the need of additional experimentation. Results demonstrate high prediction accuracy, hence facilitating the estimation of the monomer yield with respect to process settings for experiments that have yet to be performed.

Finally, a surrogate–DEM modeling approach was investigated to address the computational challenges associated with expensive high-fidelity simulations. The DEM process variables (milling frequency and ball-to-volume ratio) were translated to DEM outputs (kinetic energy and collision frequency), which were then used as inputs in the proposed model. By integrating the mechanistic DEM data into a more data-driven framework, the computational time required for the final solution was significantly reduced. This enhanced efficiency enables the use of the low-fidelity model in applications that require fast solutions such as control or flowsheet optimization.

## Supporting Information Available

High-speed videos (ZIP)
Validation of the DEM model; experimental methodology and results (PDF)

## Abbreviations

PET: poly(ethylene terephthalate)
Na_2_TPA: disodium terephthalate
EG: ethylene glycol
DEM: discrete element method
MC: mechanochemistry
S.S.: stainless-steel
CV: computer vision
SM: surrogate model
LR: linear regression
RF: random forest
