# Supplementary material for: Modeling Mechanochemical Depolymerization of PET in Ball-Mill Reactors Using DEM Simulations
Source: ACS Sustain Chem Eng. 2024 Jun 4;12(24):9003–17. doi: 10.1021/acssuschemeng.3c06081 (PMC11187622; doi:10.1021/acssuschemeng.3c06081)
Supplement: Supplementary file 2 — sc3c06081_si_003.pdf [file sc3c06081_si_003.pdf]

Supporting Information

Modeling mechanochemical depolymerization of PET in ball mill reactor using DEM simulations

Elisavet Anglou[†], Yuchen Chang[†], William Bradley[†], Carsten Sievers[†‡], Fani Boukouvala[†*]

[†]School of Chemical & Biomolecular Engineering, Georgia Institute of Technology, Atlanta, GA, 30332, USA

[‡]Renewable Bioproducts Institute, Georgia Institute of Technology, Atlanta, GA, 30332, USA

* Corresponding author: Fani Boukouvala fani.boukouvala@chbe.gatech.edu

This PDF file includes: 14 pages, 3 Figures, 4 Tables, Supporting Text and Supporting References

1 Validation of the DEM model

1.1 High-speed video analysis

To validate the developed Discrete Element Method (DEM) model, we utilized the velocity and collision data extracted from the recorded experiments using our computer vision algorithm that tracks the balls as they move during milling. A comprehensive list of properties for the recorded experiments is provided in Table S1.

.

Table S1: Operating conditions of recorded experiments. Stainless steel balls are utilized for all experiments performed.

Frequency (Hz)	Ball diameter (mm)
22.5	20.6
25.0	20.6
27.5	20.6
30.0	20.6
22.5	17.5
25.0	17.5
27.5	17.5
30.0	17.5

1.2 Discrete element method model velocity data

The DEM simulation data are sampled at intervals of 0.001 s. Because of that, most of the data are concentrated at the peaks of each of the sinusoidal functions and are not linearly distributed across the sample space as depicted in Figure S1(a). To address this data sparsity challenge, the DEM data are interpolated under the assumption that the kinematic behavior of the grinding ball will not change (e.g., the ball will continue to move towards the same direction). The interpolation is executed using the scipy.interpolate.interp1d package. Subsequently, the Savitzky–Golay filter is applied via the scipy.signal.savgol_filter package, to ensure that a uniform distribution of data points in the peak and off-peak regions is maintained. For all of the DEM simulations, the velocity data are processed after 0.2 seconds to ensure that steady state conditions have been reached. A visual representation of the DEM data as extracted from the simulation along with their corresponding interpolated versions are depicted in Figure S1(a) while the experimental data versus the DEM post-processed data are compared in Figure S1(b).

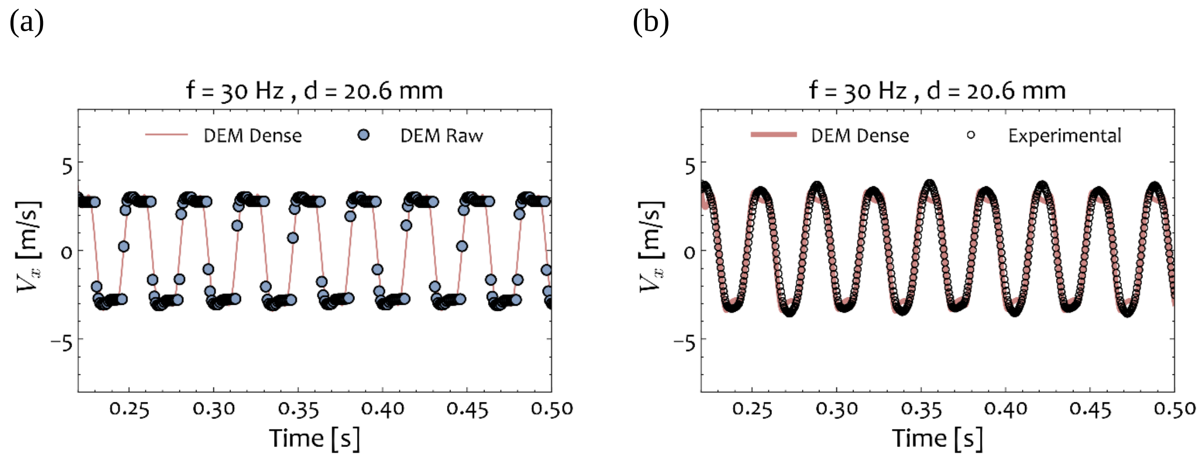

Figure S 1**:** (a) A visual representation of the DEM data as extracted from the simulation versus the interpolated data. (b) The DEM data versus the experimental data as extracted from the

computer vision algorithm. The simulation data start after 0.2 seconds of simulation time to ensure that steady state conditions are reached.

1.3 Motion experiments with powder particles

In the present work, the influence of powder on the dynamics of the milling is neglected. Assuming that the powder reactants are made up of numerous small particles with size r on the millimeter scale or less, they can be regarded as a fluidized phase characterized by number density n. When n is large enough and individual particles are dense enough, a wave of many particles may impede the motion of a ball. Suppose the particles within a thin shell of volume $4\pi R^2 \Delta R$ around a ball of radius R causes a resistance to the motion of the ball along some trajectory with $\Delta R = r$. We suggest that when the ratio α of the mass of this shell of particles to the mass of the ball is much less than 1, then the motion of the ball will not be affected by powder, and thus our neglect of powder dynamics in current experiments is justified. In terms of a ball with radius R and density ρ_B moving against a fluidized powder with number density n, density ρ and size r, α is given by:

$$\alpha = \frac{4\pi n \rho r^4}{\rho_B R} \qquad \text{Equation (S1)}$$

For our system, $\rho_B \sim 7.7$ g/cm^3 for stainless steel, $R \approx 1$ cm, $\rho \sim 1.6$ g/cm^3 (approximated as arithmetic average of PET plus NaOH), $r \sim 0.05$ cm, and n can be estimated using r and ρ over the total mass of reactants $m = 1.5$ g together with reactor volume $V_R \sim 20$ cm^3:

$$n = \frac{m}{\frac{4}{3}\pi r^3 \rho V_R} \sim 90 \text{ cm}^{-3} \qquad \text{Equation (S2)}$$

With these values we may calculate $\alpha = 1.5 \cdot 10^{-3}$, which proves that particles have a negligible effect on the motion of the milling balls during normal operation and may be safely disregarded in

motion tracking experiments. The above was also observed experimentally in which the kinematic behavior of the grinding ball was not significantly affected by the presence of PET powder particles at the specific fill-levels of experimentation compared with the main set of experiments used throughout this study. A milling experiment with PET particles and one grinding ball of d=20 mm at a milling frequency of 30 Hz is uploaded.

2 Experimental Methodology and Results

2.1 Chemicals

Poly(ethylene terephthalate) (PET) pellets were supplied by PolyQuest Inc. Sodium hydroxide (>97%) and methanol (>99.9%) were purchased from Sigma-Aldrich. Disodium terephthalate (99+%) and phosphoric acid (85% solution in water) were purchased from Alfa Aesar. These chemicals were used as received without further purification.

2.2 Mechanochemical Reactions

The procedure for PET depolymerization reactions was adapted from Tricker,[1] using a MM400 shaker mill, reinforced steel reactors and grinding spheres all manufactured by Retsch (subsidiary of Verder Scientific). In a typical experiment, 1.0 g of PET, 0.42 g of NaOH (2.1 molar equivalents relative to PET) and one 20 mm diameter grinding sphere are loaded under ambient atmosphere into a 25 mL reactor with a pill-shaped interior volume. The sealed reactor was mounted onto the ball mill and milling was conducted for a specific amount of time at a specific shaking frequency. Separate experiments are performed at each time point to avoid any interference of the reaction by opening the vessel or stopping the milling. After milling, the reaction products (powders and waxes) are collected for characterization under ambient atmosphere.

2.3 Measurement of Monomer Yields

Yields of disodium terephthalate (Na_2TPA) were determined using high performance liquid chromatography (HPLC) on an Agilent 1260 Infinity Series instrument equipped with a 15 cm

Poroshell 120 SB-C18 column and UV lamp detector set to 242 nm. The eluent used in the analysis was a mixture of 0.1 wt% $H_3PO_{4(aq)}$ and methanol programmed in gradient flow from 70% of 0.1 wt% $H_3PO_{4(aq)}$ - 30% methanol to 10% of 0.1 wt% $H_3PO_{4(aq)}$ and 90% methanol. The instrument was calibrated using terephthalic acid (TPA), which gives identical response to Na_2TPA on the UV detector.

To prepare an HPLC sample from reaction products, the 1.0-1.2 g of solids collected from an experiment was weighed and placed inside a 50 mL centrifuge tube. 50 mL of DI water was added to this tube, and then the tube was vortexed and sonicated for 30 minutes to dissolve soluble components of the solids (Solution 1) and stored overnight for insoluble residues to sediment completely. Next, 10 µL of Solution 1 was diluted with 1 mL DI water in a 2 mL centrifuge tube (Solution 2). 10 µL of solution 2 was further diluted with 700 µL of 0.1 wt% $H_3PO_{4(aq)}$ and 300 µL of methanol. Finally, this solution was passed through a 0.2 µm PTFE syringe filter into a 2 mL HPLC sample vial for analysis.

It is acknowledged that since sonication is also a type of mechanochemistry, the use of sonication to aid dissolution of analytes (Na_2TPA + EG) comes with the possibility of inducing additional hydrolysis in unreacted residual PET, and thereby introduce bias into the conversion data. To assess the significance of this possibility, Solution 1 in this study was compared to the reaction conditions in a recent related study by Chen et al., [2] which used NaOH at 1 mol/L and reaction temperatures of 50°C or more together with cosolvents to achieve good reactivity of insoluble PET pellets. Meanwhile the maximum possible concentration of NaOH in Solution 1 was calculated to be 0.02 mol/L, and the solution was purely aqueous so wetting and swelling effects on PET which aid the hydrolysis reaction were minimized. Even during bath sonication, insoluble PET particles and fragments were observed to sediment out of the dispersion of reaction products in the solution.

Therefore, low concentration of alkali and extremely unfavorable mass transfer conditions are deemed sufficient to render additional hydrolysis of unreacted PET during sonication insignificant.

Product yield was defined as the ratio of the moles of Na_2TPA (monomer) to the theoretical moles of PET at the start of reaction (assuming a molar mass of 192 g/mol) (Equation S3).

$$Y_{monomer} = \frac{n_{monomer,f}}{n_{PET,0}} \qquad \text{Equation (S3)}$$

2.4 Results of Mechanochemical PET Depolymerization Progression

The mechanochemical hydrolysis depolymerization of PET pellets was studied under the reaction scheme presented in Figure S2. The resulting yield of monomers was calculated via high-performance liquid chromatography (HPLC). Depolymerization kinetics were further explored by investigating the effect of the operating frequencies and ball mass on the achieved depolymerization yield. Milling experiments were performed in a 25 mL stainless steel vessel using one steel grinding ball with diameter of 20 mm at frequencies ranging from 25 to 30 Hz.

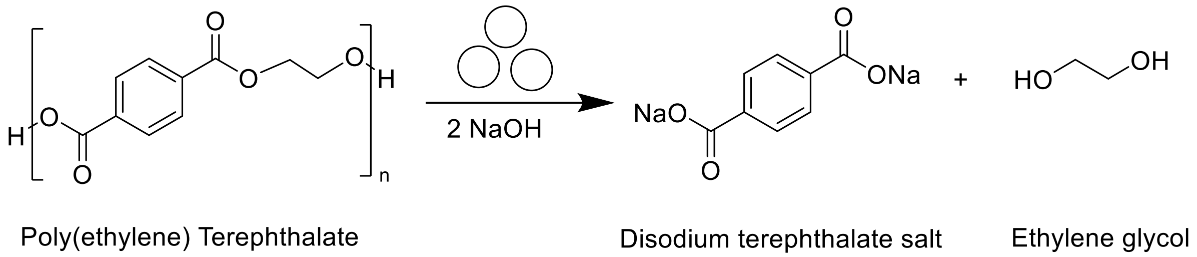

Figure S2: Alkaline hydrolysis of poly(ethylene terephthalate) (PET)

Initially, the reactant materials (PET and NaOH) are added into the reaction vessel in the form of a fine white powder (Figure S3 (a)). The monomer yield was observed to progress linearly with time until reaching an inflection point of sharp increase in yields, coinciding with the

transformation of the powder into a homogenous, sticky waxy phase (Figure S3 (b)). Samples collected in the vicinity of the inflection point where the "phase transition" of the reactant materials occur consist of a hybrid mixture of power and wax. The precise timing of when this phase transition occurs is challenging due to the rapid progression of the depolymerization reaction in that region. Complete depolymerization was achieved for all operating conditions at different times.

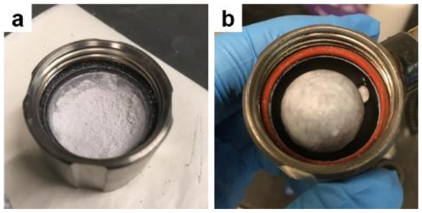

Figure S3: PET pellets milled with NaOH at (a) 10 min and (b) 20 min. The ball pictured is 20 mm in diameter while the volume of the vessel is 25 mL. Reprinted with permission from Tricker et al.[1]. Copyright 2022 American Chemical Society

The yield of Na2TPA was observed to increase with milling frequency for a given reaction time. At 30 Hz, full depolymerization was achieved in 20 min, whereas at 27.5 Hz, 100% monomer yield was reached after 25 min. At 25 Hz, depolymerization was completed after milling for 40 min. In addition, the transition from the powder to wax phase occurred at different time points for all the samples. The range of operating frequencies was selected to achieve full depolymerization at reasonable times. The experimental data depicted in Table S2-S4 (plotted in Figure 10) were utilized for the purpose of validating the modeling framework proposed in this work. The phase of the collected sample is indicated for all the data points.

Table S 2: Time (in minutes) vs. Yield data utilized in developing the link between MC experiments and DEM data for $f_{operating}$ = 30 Hz and d_{ball} = 20 mm.

Frequency = 30 Hz		
Time (min)	Yield (%)	Phase
2.2	6.2	Powder
4.8	16.1	Powder
7.4	25.0	Powder
9.9	28.1	Powder
12.3	43.0	Powder
14.8	76.4	Powder-Wax
17.2	96.9	Wax
19.8	97.1	Wax
22.4	97.1	Wax
24.7	100.0	Wax

Table S 3: Time (in minutes) vs. Yield data utilized in developing the link between MC experiments and DEM data for $f_{operating}$ = 27.5 Hz and d_{ball} = 20 mm.

Frequency = 27.5 Hz		
Time (min)	Yield (%)	Phase
4.9	10.1	Powder
10.0	22.1	Powder
14.6	38.6	Powder
16.0	53.2	Powder-Wax
18.0	83.4	Powder-Wax
19.7	93.2	Powder-Wax
24.8	99.9	Wax

Table S 4: Time (in minutes) vs. Yield data utilized in developing the link between MC experiments and DEM data for $f_{operating}$ = 25 Hz and d_{ball} = 20 mm.

Frequency = 25 Hz		
Time (min)	Yield (%)	Phase
4.8	6.2	Powder
9.9	16.0	Powder
15.0	27.7	Powder
20.0	39.3	Powder
23.0	50.7	Powder-Wax
25.4	51.4	Powder-Wax
29.9	62.1	Powder-Wax
34.8	99.1	Wax
39.8	100.0	Wax

REFERENCES

(1) Tricker, A. W.; Osibo, A. A.; Chang, Y.; Kang, J. X.; Ganesan, A.; Anglou, E.; Boukouvala, F.; Nair, S.; Jones, C. W.; Sievers, C. Stages and kinetics of mechanochemical depolymerization of poly (ethylene terephthalate) with sodium hydroxide. *ACS Sustainable Chemistry & Engineering* 2022, *10* (34), 11338-11347.

(2) Chen, H.; Hu, H. Solvent System with Improved Hydroxide Reactivity for Mild and High-Efficiency PET Alkaline Hydrolysis. *Industrial & Engineering Chemistry Research* 2023, *62* (33), 12925-12934.
